# Category-Based Attention Facilitates Memory Search

**DOI:** 10.1523/ENEURO.0012-24.2024

**Published:** 2024-02-23

**Authors:** Linlin Shang, Lu-Chun Yeh, Yuanfang Zhao, Iris Wiegand, Marius V. Peelen

**Affiliations:** ^1^Donders Institute for Brain, Cognition and Behaviour, Radboud University, Nijmegen 6525 GD, The Netherlands; ^2^Department of Mathematics and Computer Science, Physics, Geography, Mathematical Institute, Justus-Liebig-University Gießen, Gießen 35392, Germany; ^3^Department of Cognitive Science, Johns Hopkins University, Baltimore, Maryland 21218

**Keywords:** attentional template, categorical similarity, memory search, object perception, top-down modulation

## Abstract

We often need to decide whether the object we look at is also the object we look for. When we look for one specific object, this process can be facilitated by feature-based attention. However, when we look for many objects at the same time (e.g., the products on our shopping list), such a strategy may no longer be possible, as research has shown that we can actively prepare to detect only one or two objects at a time. Therefore, looking for multiple objects additionally requires long-term memory search, slowing down decision-making. Interestingly, however, previous research has shown that distractor objects can be efficiently rejected during memory search when they are from a different category than the items in the memory set. Here, using EEG, we show that this efficiency is supported by top-down attention at the category level. In Experiment 1, human participants (both sexes) performed a memory search task on individually presented objects from different categories, most of which were distractors. We observed category-level attentional modulation of distractor processing from ∼150 ms after stimulus onset, expressed both as an evoked response modulation and as an increase in decoding accuracy of same-category distractors. In Experiment 2, memory search was performed on two concurrently presented objects. When both objects were distractors, spatial attention (indexed by the N2pc component) was directed to the object that was of the same category as the objects in the memory set. Together, these results demonstrate how top-down attention can facilitate memory search.

## Significance Statement

When we are in the supermarket, we repeatedly decide whether a product we look at (e.g., a banana) is on our memorized shopping list (e.g., apples, oranges, kiwis). This requires searching our memory, which takes time. However, when the product is of an entirely different category (e.g., dairy instead of fruit), the decision can be made quickly. Here, we used EEG to show that this between-category advantage in memory search tasks is supported by top-down attentional modulation of visual processing: The visual response evoked by distractor objects was modulated by category membership, and spatial attention was quickly directed to the location of within-category (vs between-category) distractors. These results demonstrate a close link between attention and memory.

## Introduction

Visual object processing is modulated by top-down goals. For example, the same object evokes a stronger neural response in visual cortex when the object is a target as compared to when it is a distractor (e.g., Chelazzi et al., 1993; Bansal et al., 2014). These modulations have typically been studied in the context of attention, with a top-down attentional set (or “template”) modulating visual processing (Desimone and Duncan, 1995). Such templates can operate at different levels of the visual hierarchy, from simple visual features to high-level object categories (Battistoni et al., 2017).

While the mechanisms behind single-target detection have been extensively studied, much less is known about multiple-target detection (Ort and Olivers, 2020), even though this task is common in daily life. For example, when we are in the supermarket, we must decide whether the product we look at is one of the (possibly many) products on our memorized shopping list. This task is typically referred to as a memory search task (Sternberg, 1966), as it involves searching memory for the currently fixated item. Indeed, as memory set size (MSS) increases, responses systematically slow down, reflecting the memory search process (Wolfe, 2012).

An important difference between single-target and multiple-target detection is that observers can no longer use an attentional template-based strategy when looking for multiple targets. This is because only one or two attentional templates can be activated at a given time (Houtkamp and Roelfsema, 2006; Olivers et al., 2011; van Moorselaar et al., 2014; Ort and Olivers, 2020; Wolfe, 2021). Accordingly, the attentional template-based modulation of visual object processing, as observed for single-target detection, may be absent for multiple-target detection (i.e., memory search). This is in line with findings from memory research, showing relatively late (∼300–500 ms) electroencephalography (EEG) responses over mid-frontal and parietal electrodes reflecting recognition memory (for a review, see Rugg and Curran, 2007), rather than the earlier (150–200 ms) attentional modulation observed in single-target detection tasks (VanRullen and Thorpe, 2001; Kaiser et al., 2016).

**Figure 1.**
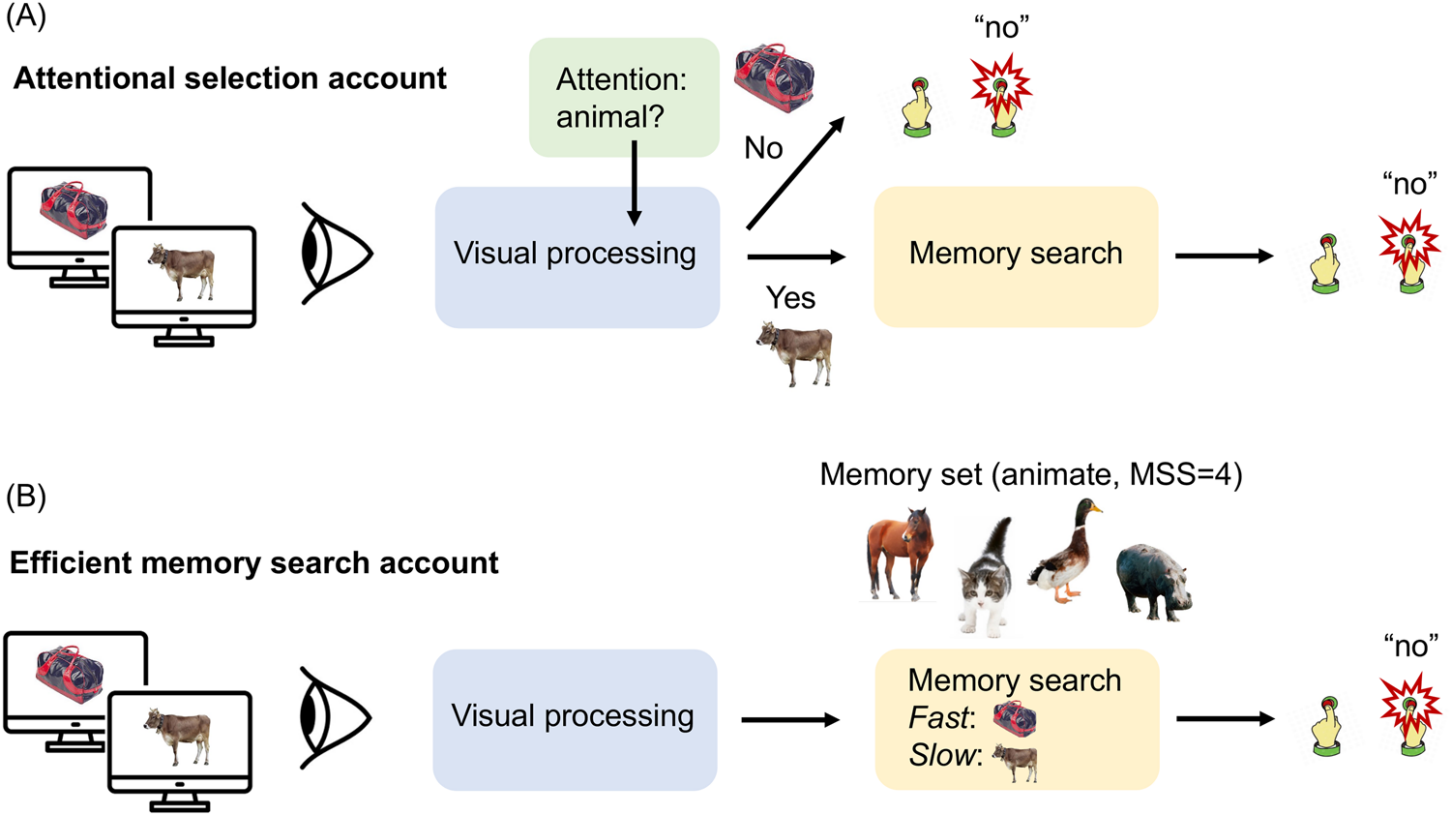
Schematic illustration of two accounts explaining category effects in memory search. The memory set in this example consists of four animals. The current study was aimed at providing evidence for category-level attentional selection in a memory search task, as illustrated in panel ***A***. On this attentional selection account, participants spontaneously attend to the category of the items in the memory set, modulating visual processing and resulting in the quick rejection of between-category distractors, largely avoiding memory search for these items. An alternative account (illustrated in panel ***B***) holds that all objects enter the memory search phase after visual object processing is completed. On this account, faster RTs for between-category than within-category distractors are explained by differences in the efficiency of the memory search process.

Interestingly, however, memory search efficiency depends on the categorical relationship between the objects in the memory set and the probe (Cunningham and Wolfe, 2012, 2014; Drew and Wolfe, 2014). If the probe (e.g., a banana) is of the same category as the items in the memory set (e.g., apple, pear, orange), search is inefficient, such that RT increases strongly with increasing MSS. However, when the probe is of a different category (e.g., an animal), search is highly efficient, such that RT increases only weakly with increasing MSS (Cunningham and Wolfe, 2012, 2014; Drew and Wolfe, 2014). Previous work has proposed that the between-category advantage in memory search is mediated by visual selective attention, with between-category distractors rejected as possible targets before entering the memory search stage (Cunningham and Wolfe, 2014). Indeed, participants may have spontaneously used the shared category of the memory items to form a category-level attentional template, thereby obviating the need to search long-term memory (Fig. 1*A*). An alternative explanation is that between-category distractors are rejected efficiently because they are represented distinctly in long-term memory (Fig. 1*B*). Behavioral results alone cannot distinguish between these explanations. However, the category-based attention explanation (Fig. 1*A*) makes specific predictions about the neural time course of processing within- versus between-category distractors, which we tested in the current study.

Here, in two experiments, we used EEG to test if, and when, visual object processing is modulated by category similarity in a memory search task. In Experiment 1, we found that the visually evoked N1 ERP component differentiated between distractors that were of the same versus a different category as the memorized items, in line with the selective attention account. In Experiment 2, we used two-object displays to show that this modulation is followed by the allocation of spatial attention toward within-category distractors. Altogether, our results show that the between-category advantage in memory search can be explained by category-level attentional selection.

## Materials and Methods

### Participants

#### Experiment 1a

Experiment 1a was run online. Forty-one participants (15 females; mean age, 23.81 years; age range, 20–35 years) were recruited from the online platform Prolific to arrive at a final sample size of 40. Twenty participants (8 females; mean age, 22.95 years; age range, 20–28 years) were assigned to the animate-category group, while 21 participants (7 females; mean age, 24.94 years; age range, 20–35 years) were assigned to the inanimate-category group. One participant in this group had to be excluded because of low accuracy (ACC) for set size 16 (<65%). All participants signed an online informed consent form and received 6 euro per hour for their participation in the experiment, which was approved by the Ethics Committee of the Faculty of Social Sciences, Radboud University Nijmegen.

#### Experiment 1b

We employed pwr [R] package to compute the sample size at the significance level of 0.05 with Cohen's *d* = 0.5 (corresponding to a medium effect size) and power = 0.8. Thirty-four participants were needed. We included 32 participants (23 females; age range, 18–30 years with Mean = 22.031 years and SD* *= 3.036) in Experiment 1b because of the Latin square design (see *Experimental design and procedure*). All participants had normal or corrected-to-normal vision. The participants gave written informed consent and received a gift card of 10 euro per hour for their participation. The study was approved by the Ethics Committee of the Faculty of Social Sciences, Radboud University Nijmegen.

#### Experiment 2

To arrive at 32 participants, as in Experiment 1b, 35 right-handed participants (11 females; age ranges from 18 to 35 years with Mean = 22.51 years and SD = 3.61) were recruited. Three participants were excluded due to missing more than 20% of trials after incorrect responses exclusion and artifact rejection. All participants had normal or corrected-to-normal vision. The participants gave written informed consent and received a gift card of 10 euro per hour for their participation. The study was approved by the Ethics Committee of the Faculty of Social Sciences, Radboud University Nijmegen.

### Stimuli

#### Experiment 1a

Stimuli consisted of full-color photographs of isolated animals (from Google Images) and inanimate objects (from Brady et al., 2008). Both categories had 30 subcategories (e.g., horse, cat, binoculars, bowl), and each subcategory consisted of 17 exemplar images, for a total of 1,020 unique images. Stimulus size was 500 × 500 pixels. The experiment was programmed with PsychoPy v2020.2.3 (Peirce et al., 2019) and was hosted on Pavlovia.

#### Experiment 1b

The stimuli were the same as in Experiment 1a. The experiment was programmed with PsychoPy v2022.2.4 (Peirce et al., 2019) and ran on a 24-inch monitor (BenQ XL2420Z) with a refresh rate of 120 Hz and a resolution of 1,920 × 1,080. Participants were required to keep a distance of approximately 57 cm from the screen, and the stimuli subtended a visual angle of 4.9°.

#### Experiment 2

The stimuli were a subset of those used in Experiment 1, removing one subcategory of each superordinate category for a total of 986 full-color images of unique isolated objects. Stimuli were presented on a white background with a visual angle of 4°. The experiment was programmed with PsychoPy v2022.2.5 (Peirce et al., 2019) and was presented on a 24-inch monitor (BenQ XL2420Z) with a refresh rate of 120 Hz and a resolution of 1,920 × 1,080.

### Experimental design and procedure

#### Experiment 1a

**Figure 2.**
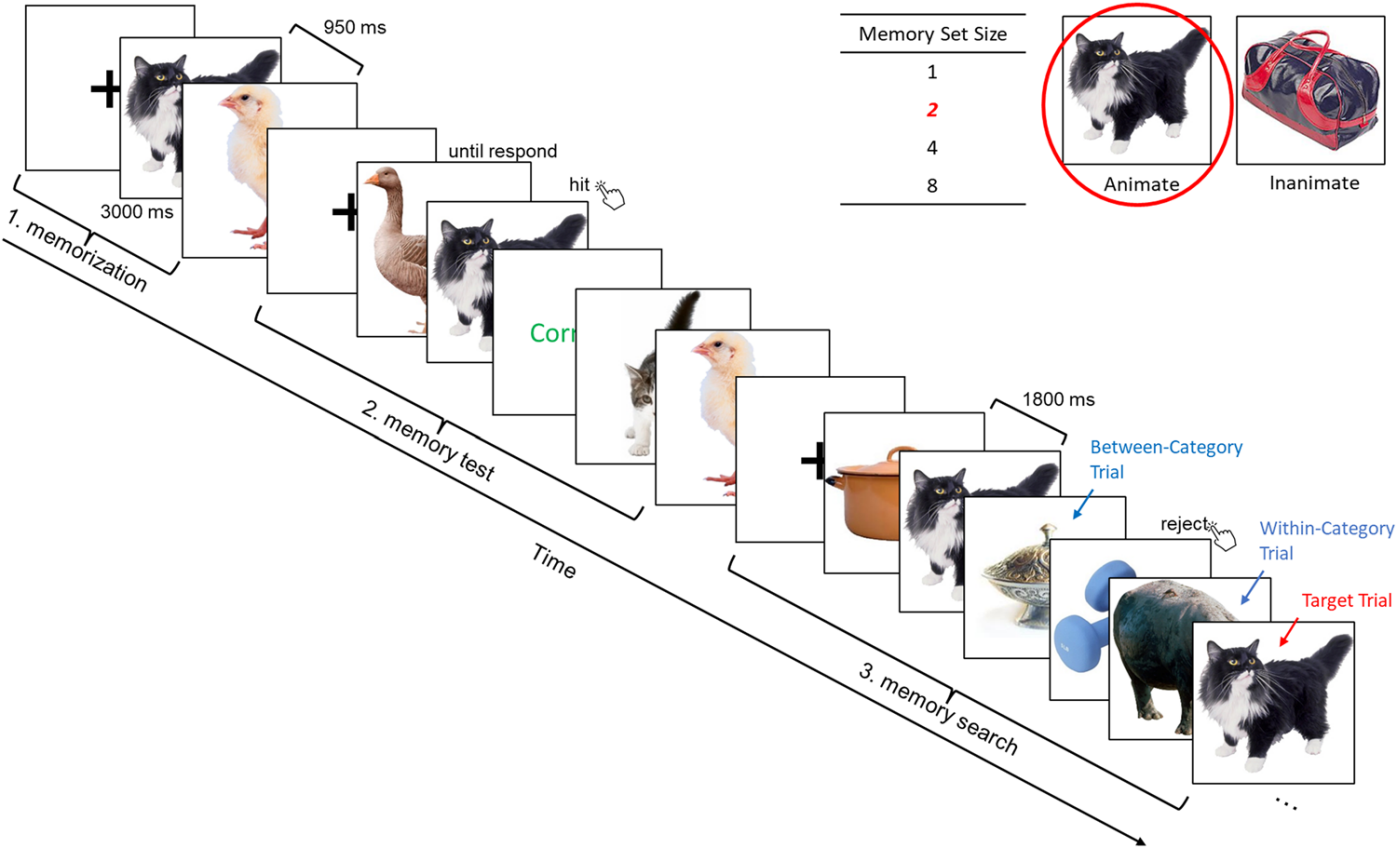
Illustration of the three phases in each block of Experiments 1a and b. The example presents an animate block with two targets (i.e., MSS 2).

The experimental design followed a 2 (animate/inanimate category group; between-subjects) × 2 (within/between category; within-subjects) × 5 (MSS 1/2/4/8/16; within-subjects) mixed factorial design. Category group was manipulated between subjects, such that each participant remembered either animate or inanimate objects in all blocks of the experiment. MSS was blocked, with one block for each MSS, for a total of five blocks per participant. Block order was randomized. Each block consisted of the following three phases (Fig. 2):

1. In the first phase (memorization), participants memorized the objects belonging to the memory set in that block. Object images were randomly assigned to the memory set, with the constraint that each image in the set came from a different subordinate category (e.g., horse, cat). All images in the memory set were new to the participant. In each trial, a central fixation cross appeared for 800 ms as a prompt, followed by an object presented in the center of the screen on a white background, one at a time, for 3,000 ms with an inter-stimulus interval (ISI) of 950 ms. Participants were instructed to memorize the objects without giving a response.
2. In the second phase (memory test), participants again viewed the objects but now had to indicate, with a button press, whether the object belonged to the memory set (press “z”) or not (press “m”). This task was self-paced. Nontarget objects were randomly drawn from the same sub-ordinate categories as the target objects (e.g., a different cat). Half of the objects were targets and half were not, presented in random order without repetition. Participants had to be at least 80% correct on two subsequent tests to be able to proceed to the next phase of the experiment. If they did not meet this criterion, they would repeat Phase 1.
3. In the third and main phase (memory search) of each block, participants performed a speeded old/new recognition task, deciding for each object whether or not it was part of the memory set. This phase consisted of 60 trials, presented in random order. Twenty percent of the trials (12 trials) showed an image selected from the memory set, while the remaining 48 trials were target-absent trials. Of these target-absent trials, 24 belonged to the animate category and 24 belonged to the inanimate category. Therefore, depending on the category group the participant was assigned to, these could be either within- or between-category distractors (relative to the memory set). In each trial, a central fixation cross appeared for 800 ms as a prompt, followed by an object presented in the center of the screen on a white background for 200 ms with an ISI of 1,800 ms. Participants had to indicate whether the object belonged to the memory set within 2,000 ms. The target images were randomly drawn from the memory set, such that these could repeat within a block. However, all the nontarget images were unique across the whole experiment.

#### Experiment 1b

Memory search. The memory search design generally followed the design of Experiment 1a with minor adjustments. To simplify the experimental procedure, set size 16 was removed. Furthermore, unlike Experiment 1a, the category (animate or inanimate) was manipulated within participants (i.e., participants memorized the targets from both animate and inanimate categories). As in Experiment 1a, each MSS was designed as a block; there were therefore 8 blocks in total in the memory task. A Latin square design was used to order the four MSS blocks, with category order within MSS randomly determined. Unlike Experiment 1a, the fixation cross was always presented except during the stimulus presentation, and the ISI in the memory search phase was jittered between 1,800 and 2,300 ms. Other procedures were identical to Experiment 1a (Fig. 2).

Visual oddball task. A visual oddball task was included to measure visually evoked response patterns to animate and inanimate objects without a memory search task. These data were used to train an animate/inanimate classifier, ensuring that classifier training was done on independent data. In each run, 50 animate and 50 inanimate objects were shown for 200 ms, one by one, with an ISI of 1,800–2,300 ms, in random order. The 100 objects were randomly selected from the same stimulus pool as used for the memory search task. Objects that were selected for the oddball task were not selected for the memory search task. In addition to the objects, there were ten two-digit numbers that were randomly interspersed. Participants pressed a button when seeing one of these numbers. In total, participants performed the oddball task three times. These runs were preceded and followed by two memory search blocks.

EEG acquisition and pre-processing*.* Scalp EEG signals were recorded with a customized 64-channel active electrode actiCAP system with 500 Hz sampling rate. AFz served as ground electrode, and TP9 placed on left mastoid as a reference electrode. FT9/FT10 and Fp1/Fp2 were reset to left/right and up/down eye movement recorder. Impedance of all the electrodes was kept below 20 kΩ. The EEG data were pre-processed in Python 3.10 using custom code adapted from the MNE toolbox (Gramfort, 2013). All the data were bandpass filtered (0.1 and 40 Hz) and resampled to 250 Hz. Each trial epoch was segmented from −200 to 800 ms relative to the onset of the object. Only epochs with correct responses were included in further analyses. Then, independent component analysis (ICA) was performed for each subject to remove components of eye movements and blinks. Finally, the ICA-corrected data were re-referenced to the average of all channels and were baseline corrected by subtracting the mean activity from −200 to 0 ms.

#### Experiment 2

**Figure 3.**
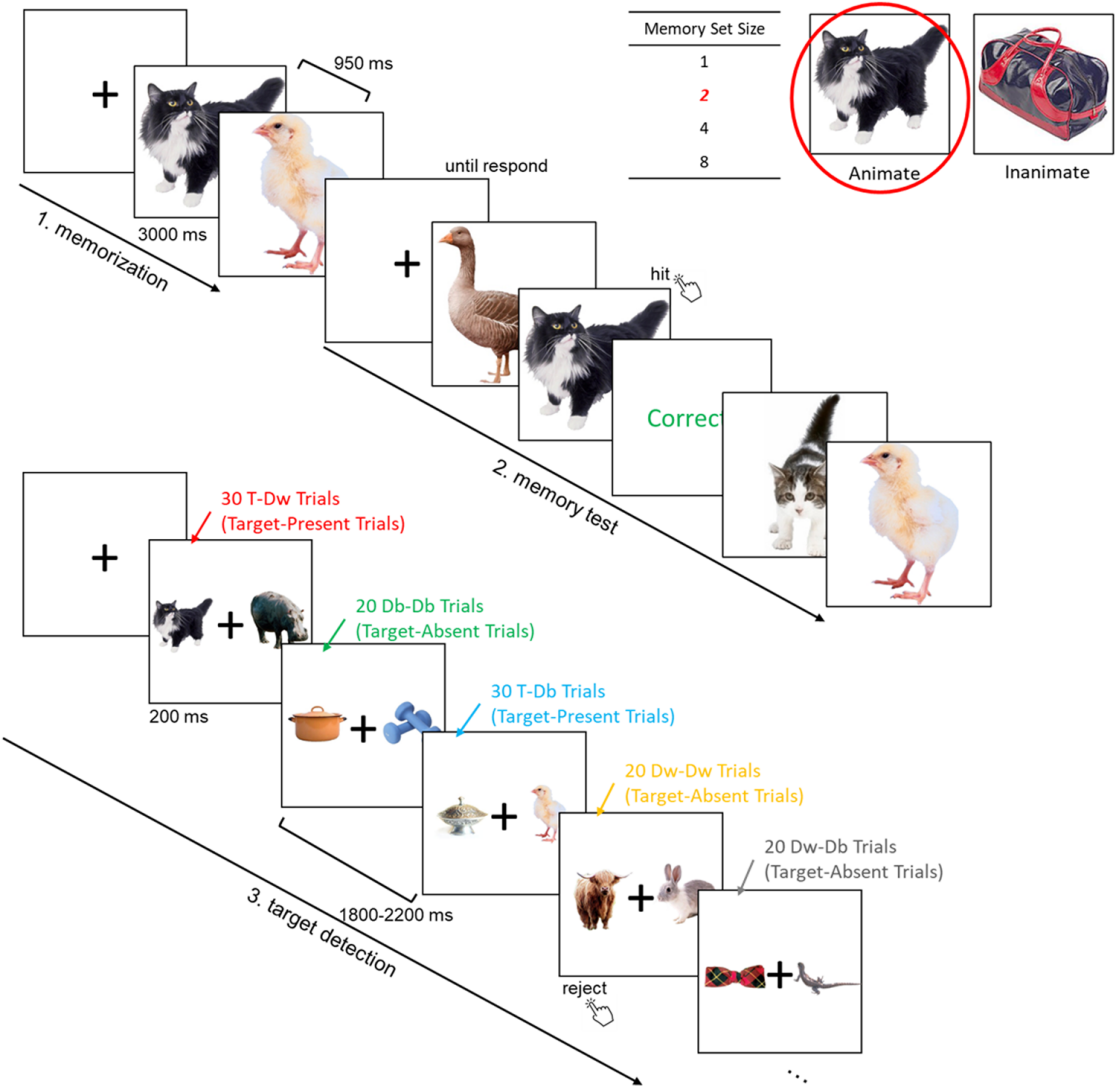
Illustration of the procedure of Experiment 2. The example presents an animate block with two targets (i.e., MSS 2). For target-present trials, a target shown with a within-category distractor condition was abbreviated as T-Dw, a target with a between-category distractor as T-Db; for target-absent trials, trials showing one distractor of the target (i.e., within) category together with one distractor of the other (i.e., between) category was abbreviated as Dw-Db, trials where both distractors were within category as Dw-Dw. Finally, trials where both distractors were between category were abbreviated as Db-Db. Our main analyses focused on T-Dw, T-Db, and Dw-Db conditions, as we hypothesized lateralized responses in these conditions.

As in Experiment 1b, Experiment 2 manipulated category (animate/inanimate) and MSS (1/2/4/8) within subjects, resulting in eight blocks per participant. Block order was randomized. Each block again consisted of three phases (Fig. 3).

Unlike Experiment 1b, the third phase now consisted of a memory search task in which participants were presented with a pair of object images. Participants decided, as quickly as possible, whether the image pair included one of the memorized objects. Each block included a total of 120 trials, presented in random order. In target-present trials (50%), the target was presented together with a within- or a between-category distractor (Fig. 3). In target-absent trials (50%), three combinations of two distractors (two within-category distractors, two between-category distractors, one within- and one between-category distractors) were presented with equal probability (Fig. 3). Category (animate/inanimate) and target location (left/right visual field) were counterbalanced for each combination. At the beginning of each trial, a black fixation cross appeared for 500 ms, followed by one of the five combinations randomly presented for 200 ms, with a jittered inter-trial interval between 1,800 and 2,200 ms, during which participants needed to press the up arrow key or down arrow key to indicate whether the image pair contained a target or not. Participants were instructed to blink only after making a response.

EEG acquisition and pre-processing. The EEG acquisition system and the pre-processing were exactly the same as Experiment 1b. However, trials with correct responses were segmented from −200 to 500 ms relative to the onset of the image pair. Eye movements and other artifacts (e.g., blinks) were removed based on visual inspection (Yeh and Peelen, 2022) rather than using ICA, considering the cortical overlap and functional relationship between saccades and spatial attention shifts (Kowler et al., 1995), which could lead to distortion of the N2pc signal. After that, the clean data were re-referenced to the average of all channels. Baseline correction was from −200 to the stimulus onset. All the pre-processing above was completed in Python 3.11 using the MNE toolbox (Gramfort, 2013).

### Statistical analyses

#### Experiment 1a

All analyses focused on the reaction time (RT) to target-absent trials (80% of trials) in the memory search task, of which each image was presented only once. Only correct responses and RTs above 200 ms were included in further analyses. Furthermore, for each participant, RTs beyond 3 standard deviations (SD) from the condition mean were excluded. Under this criterion, a total of 0.260% data points were excluded from further analyses. RT was analyzed in a three-way mixed-design analysis of variance (ANOVA) with one between- (animate-/inanimate-category group) and two within-subjects factors (within-/between-category condition; MSS 1/2/4/8/16). The Greenhouse–Geisser correction was applied to adjust for lack of sphericity (Jennings and Wood, 1976), and only corrected degrees of freedom and *p*-values are reported. Because the three-way interaction was not significant, *F*_(4, 152)_ = 1.228, *p* = 0.277, *η_P_*^2^ = 0.033, we collapsed the data across animate and inanimate groups in all subsequent analyses. Accuracy was >92% in all conditions. The result pattern of accuracy across conditions was in line with the RT results (data not shown).

#### Experiment 1b

The exclusion criteria of behavioral data were identical to those of Experiment 1a. In total, 1.139% of data points were removed. Only correct responses in target-absent trials were included for both behavioral and EEG analysis. Accuracy was >98% in all conditions and the pattern of accuracy across conditions supported the RT results (data not shown).

ERP analyses. The ERP analysis focused on P1, N1, and P2 components over posterior electrodes. We first visually inspected the visually-evoked ERP waveform, both at the group level and for each individual participant. Averaging across participants and conditions, the following 12 posterior electrodes showed a strong visually-evoked ERP consisting of P1, N1, and P2 components: P5/P6, P7/P8, PO3/PO4, PO7/PO8, PO9/PO10, and O1/O2. These components were also visible in each individual participant. The time window for each component was defined based on the peak range among the participants (Robinson et al., 2015). P1 peak was observed between 100 and 160 ms after stimulus onset. N1 and P2 peaks were from 160 to 200 ms and from 200 to 300 ms, respectively.

Two-way repeated-measures ANOVA (MSS 1/2/4/8 × within-/between-category conditions) was employed to test for differences in the mean amplitudes of the 12 posterior electrodes across MSS and category conditions, separately for the P1, N1, and P2 components. The Greenhouse–Geisser correction was applied for adjusting for lack of sphericity (Jennings and Wood, 1976), and only corrected degrees of freedom and *p*-value are reported. Then, cluster-based nonparametric permutation tests (Maris and Oostenveld, 2007) were employed to further examine the time courses of the main effects and interaction with 4 ms resolution from 100 to 300 ms; the range that included the three ERP components.

Decoding analyses. Based on the pattern across the 12 electrodes, a linear support vector machine (SVM) was employed to conduct cross-task decoding analysis. The visual oddball task data was used to train an animacy decoder, which was used to decode the object categories in the memory search task between 0 and 600 ms, separately for each participant. Temporal resolution was down-sampled to 100 Hz. The area under the receiver operator characteristic curve (AUC) was employed to evaluate the performance on classification, which referred to the probability to distinguish positive and negative classes. As a classification metric, it is independent from the classifier threshold and more robust for imbalanced classes than classification accuracy (Treder, 2020).

#### Experiment 2

The exclusion criteria for removing trials based on behavioral responses were identical to those of Experiment 1. In total, 1.471% of data points were excluded from further analysis. The accuracy was >91% in all conditions, and the pattern of accuracy across conditions supported the RT results (data not shown).

ERP analyses. ERP analyses focused on the amplitude of the N2pc component, which was defined by the time window of 200–299 ms (Luck and Hillyard, 1994b; Yeh and Peelen, 2022) at two electrode sites PO7/8 (Mazza et al., 2007; Eimer and Kiss, 2008; Kiss et al., 2008; Burra and Kerzel, 2013; Stoletniy et al., 2022). For target-present trials, differences in the N2pc (contralateral-ipsilateral responses) were tested in a two-way repeated-measures ANOVA (MSS 1/2/4/8 × T-Dw/T-Db category). Finally, cluster-based nonparametric permutation tests (Maris and Oostenveld, 2007) were adopted to test the time courses of the main effects and interaction with 4-ms resolution from 100 to 400 ms.

## Results

### Experiment 1a

Experiment 1a was a behavioral study aimed at replicating previous findings of category effects in memory search (e.g., Drew and Wolfe, 2014) but now using a paradigm that would be suitable to use with EEG (Experiment 1b). To avoid differential repetition effects across conditions (Nosofsky et al., 2014a), we measured behavioral responses to individually presented distractor objects, with distractor objects making up 80% of trials. Each distractor image was only shown once. We asked: (1) whether search efficiency was modulated by the categorical similarity between the distractors and the objects in the memory set, and (2) whether RTs for distractors under these conditions would follow the typical log-linear relationship with set size (e.g., Wolfe, 2012; Drew and Wolfe, 2014).

#### Set size and category effects

**Figure 4.**
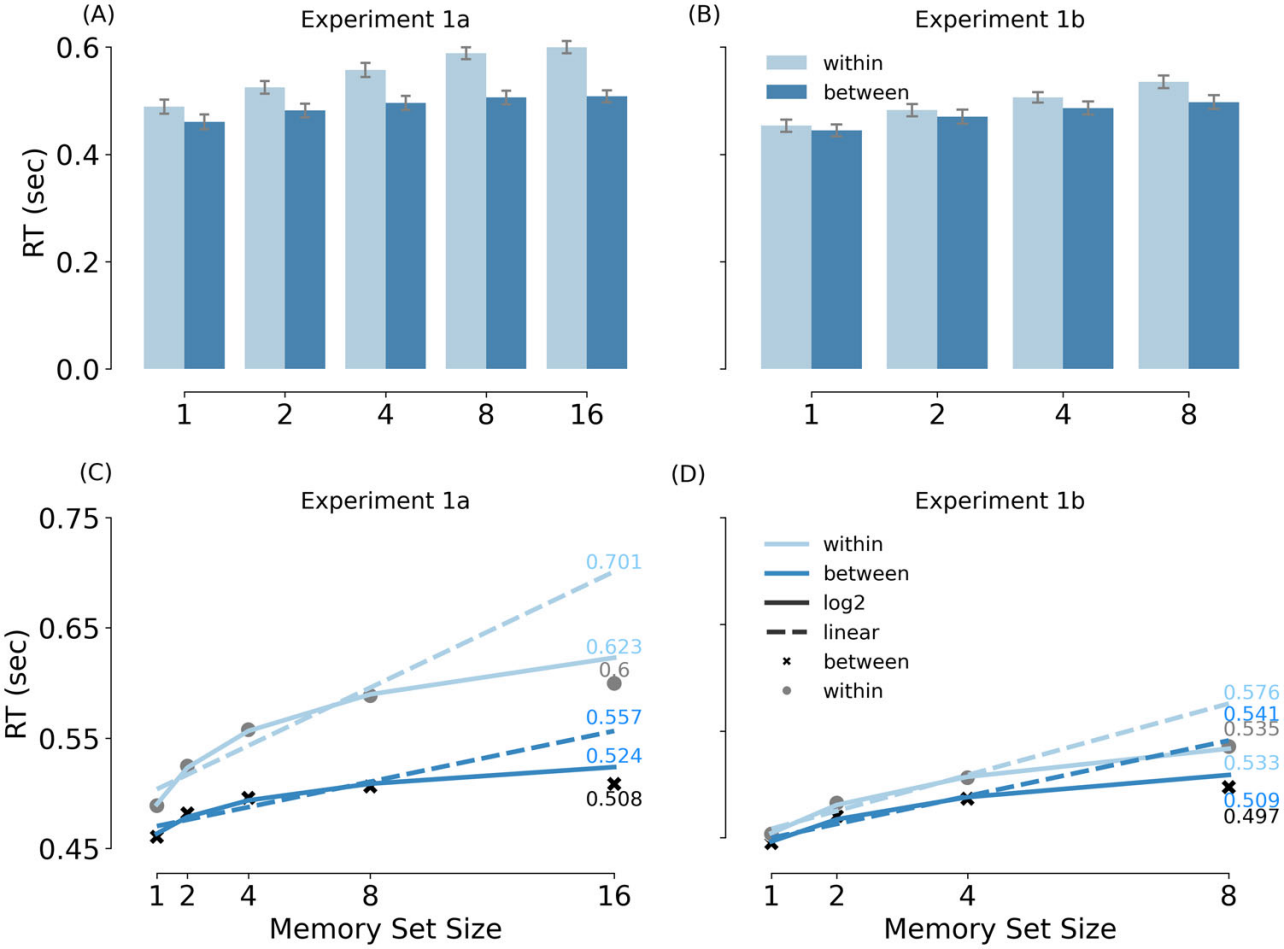
Mean RT (in sec) and fitted models in Experiment 1a and b. ***A,B***, Show the mean RTs in Experiment 1a and b, respectively. ***C*,*D***, Show the fitted models in Experiment 1a and b, respectively. The light gray dots and dark gray crosses refer to the observed data in within-category and between-category conditions. The dashed lines represent linear models, while the solid lines represent log-linear models. For all the figures, the error bars represent the standard error of the mean; the light blue represents within-category conditions while the dark blue represents between-category conditions.

A two-way repeated-measures ANOVA with RT as dependent variable and MSS (1/2/4/8/16) and category (within-/between-category conditions) as independent variables revealed significant main effects of MSS: *F*_(3.41, 133)_ = 35.521, *p* < 0.001, *η_P_*^2^ = 0.477, and category, *F*_(1, 39)_ = 320.039, *p* < 0.001, *η_P_*^2^ = 0.891. Furthermore, the interaction between MSS and category was significant: *F*_(4, 156)_ = 24.230, *p* < 0.001, *η_P_*^2^ = 0.383. As can be observed in Figure 4*A*, MSS had a stronger effect (i.e., memory search was less efficient) for within-category than between-category distractors. The simple main effect of set size was significant for both within-, *F*_(4, 156)_ = 54.442, *p* < 0.001, *η_P_*^2^ = 0.583, and between-category conditions, *F*_(4, 156)_ = 10.873, *p* < 0.001, *η_P_*^2^ = 0.218.

#### Linear versus loglinear models

The increase with MSS visibly displayed a nonlinear increase, in line with previous work (Wolfe, 2012; Drew and Wolfe, 2014). To confirm these results statistically, the RTs from set size 1–8 were used to predict the performance on set size 16 (Fig. 4*C*), following previous studies (e.g., Drew and Wolfe, 2014). The absolute error of the loglinear model prediction, compared to the observed data, was significantly smaller than the absolute error of the linear model prediction, for both within-category, *t*_(39)_ = −7.582, *p* < 0.001, *d* = 1.199, and between-category conditions, *t*_(39)_ = −5.382, *p* < 0.001, *d* = 0.851.

Finally, we fitted the log-linear model to the observed data using all five set sizes. Confirming the category × MSS interaction observed in the ANOVA, the log-linear slope coefficients for the two category conditions differed significantly, *t*_(39)_ = 9.604, *p* < 0.001, *d* = 1.519, with a steeper slope for within- than between-category distractors.

#### Summary

In this behavioral experiment, we replicated previous findings of a log-linear increase of RT with MSS (e.g., Wolfe, 2012; Drew and Wolfe, 2014). Interestingly, this was observed for distractor objects, which made up 80% of trials. Furthermore, each of these objects was presented only once, excluding the possibility that the set size effect reflected the influence of differential repetition (e.g., items repeating more often in low than high set size conditions; Nosofsky et al., 2014a,b). Most importantly for the present purpose, we found a strong category effect on search efficiency: memory search was much more efficient for distractors that were categorically dissimilar to the items in the memory set than for distractors that were of the same category as the items in the memory set (Cunningham and Wolfe, 2012, 2014; Drew and Wolfe, 2014).

### Experiment 1b

Experiment 1b adopted EEG to test when categorical similarity modulates the processing of the distractor objects. We reasoned that if the between-category advantage is driven by the (proactive) use of categorical attentional templates, this would be observed as a modulation of visual processing (150–200 ms). By contrast, if the between-category advantage is due to a more efficient search in memory (postvisual processing), no such early modulation would be observed. Accordingly, we focused our analysis on two visually evoked event-related potential (ERP) components that emerge within the first 200 ms after stimulus onset: P1 and N1. While the P1 is only modulated by spatial attention, the N1 is modulated by feature-based attention (Motter, 1994; Hopf et al., 2004; but see Zhang and Luck, 2009). Similar to feature-based attention, category-based attention was shown to modulate processing from 150 to 200 ms after stimulus onset (VanRullen and Thorpe, 2001), with better decoding of attended than unattended categories at this latency (Kaiser et al., 2016). Based on these findings, we expected that a category-based attention mechanism during memory search would similarly modulate the N1 component and increase the accuracy of object category decoding at that latency. Finally, the P2 component was also included in our analyses, based on previous studies implicating the P2 in matching perceptual inputs to memory templates (Luck and Hillyard, 1994a; Dunn et al., 1998; Lefebvre et al., 2005; Freunberger et al., 2007).

#### Behavioral results

Figure 4*B* shows the behavioral results of Experiment 1b. These results replicated the findings of Experiment 1a. There were significant main effects of MSS: *F*_(3, 93)_ = 31.591, *p* < 0.001, *η_P_*^2^ = 0.505, and category, *F*_(1, 31)_ = 91.205, *p* < 0.001, *η_P_*^2^ = 0.746. As in Experiment 1a, the interaction between MSS and category was significant: *F*_(2.34, 72.61)_ = 6.798, *p* = 0.003, *η_P_*^2^ = 0.180. Simple effects of MSS were significant in both within-category condition, *F*_(3, 93)_ = 42.176, *p* < 0.001, *η_P_*^2^ = 0.576, and between-category condition, *F*_(3, 93)_ = 14.41, *p* < 0.001, *η_P_*^2^ = 0.317. Pairwise comparisons showed significant category effects for all MSSs (*p* < 0.001), except for set size 1 (*p* = 0.175).

The linear/log2 prediction based on set sizes 1–4 demonstrated that the loglinear model was a better fit than the linear model for both within-category and between-category conditions (Fig. 4*D*), as demonstrated by a significantly smaller absolute error between predicted and observed data for the loglinear model: within category, *t*_(31)_ = −5.389, *p* < 0.001, *d* = 0.953, between-category, *t*_(31)_ = −4.244, *p* < 0.001, *d* = 0.750. The slope coefficients of these two conditions (fitting the model on all set sizes) were also significantly different, *t*_(31)_ = 3.488, *p* = 0.001, *d* = 0.617.

#### ERP results

**Figure 5.**
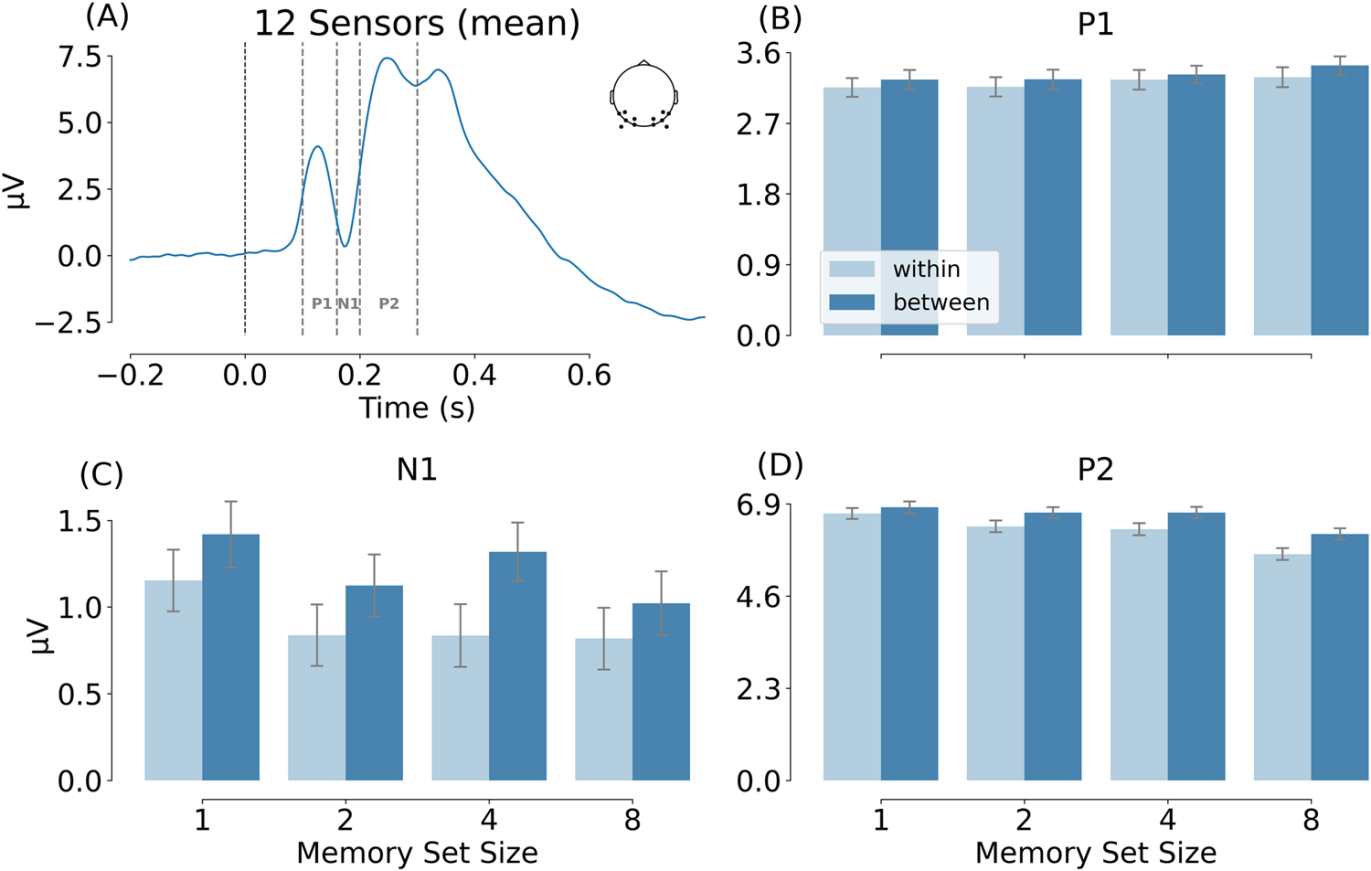
EEG results of Experiment 1b. ***A***, The mean amplitude based on 12 electrodes (P5/P6, P7/P8, PO3/PO4, PO7/PO8, PO9/PO10, and O1/O2) averaged across all conditions, illustrating the three ERP components of interest. ***B–D***, Compare the mean amplitude of the 12 electrodes in the two category and four MSS conditions.

Separate ANOVAs were run for the three components of interest (P1, N1, P2). There were no significant effects for the P1: MSS effect, *F*_(3, 93)_ = 0.629, *p* = 0.598, *η_P_*^2^ = 0.020; category effect, *F*_(1, 31)_ = 1.593, *p* = 0.216, *η_P_*^2^ = 0.049; and interaction, *F*_(3, 93)_ = 0.034, *p* = 0.992, *η_P_*^2^ = 0.001 (Fig. 5*B*). Importantly, confirming our hypothesis, the N1 showed a significant main effect of category: *F*_(1, 31)_ = 9.185, *p* = 0.005, *η_P_*^2^ = 0.229 (Fig. 5*C*). The main effect of MSS, *F*_(3, 93)_ = 2.272, *p* = 0.085, *η_P_*^2^ = 0.068, and the interaction between category and MSS, *F*_(3, 93)_ = 0.285, *p* = 0.836, *η_P_*^2^ = 0.009, were not significant. Finally, the P2 showed main effects of MSS: *F*_(3, 93)_ = 9.233, *p* < 0.001, *η_P_*^2^ = 0.229, and category, *F*_(1, 31)_ = 18.195, *p* < 0.001, *η_P_*^2^ = 0.370 (Fig. 5*D*). The interaction between category and MSS was not significant: *F*_(3, 93)_ = 0.650, *p* = 0.585, *η_P_*^2^ = 0.021.

**Figure 6.**
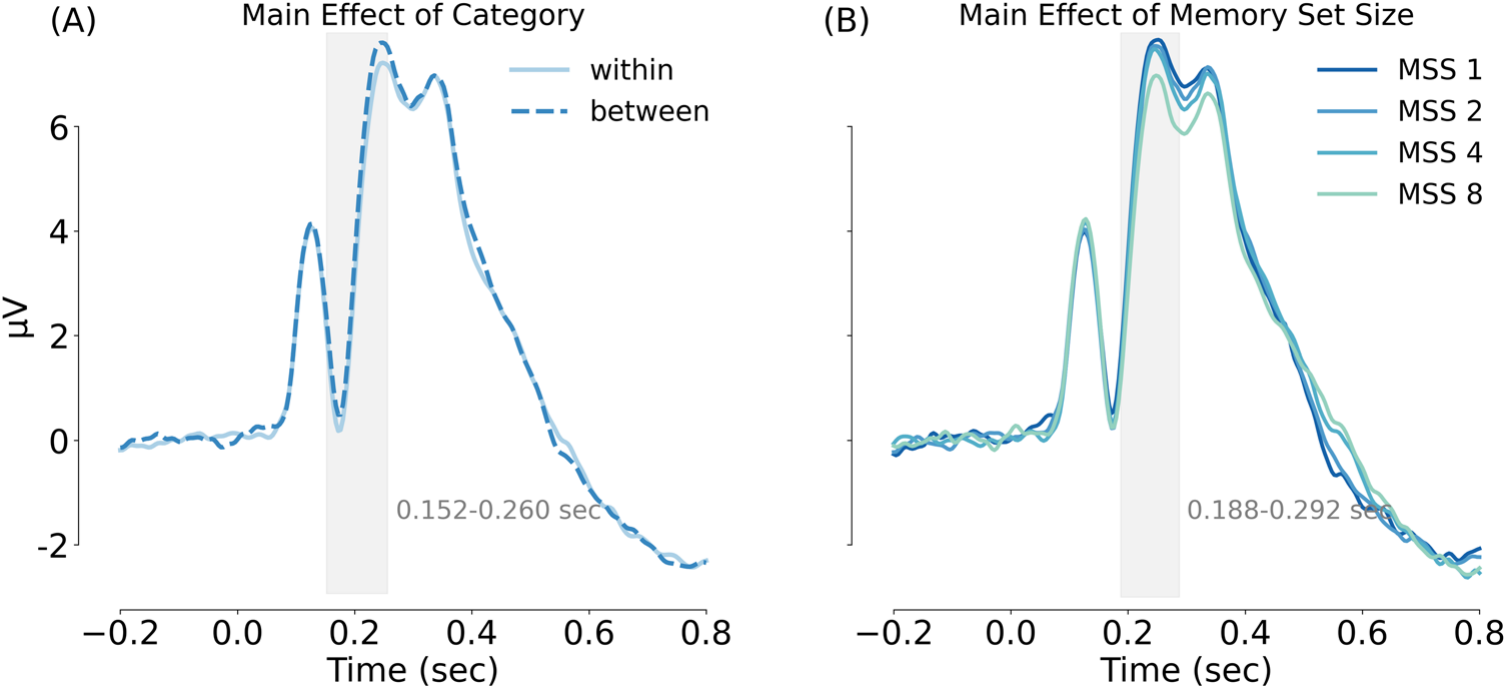
Cluster-based analyses of Experiment 1b. ***A***, Main effect of category. The light blue solid line represents the within-category condition, while the dark blue dashed line represents the between-category condition. ***B***, Main effect of MSS. The blue color from dark to light refers to set size 1, 2, 4, and 8, respectively. The gray bars indicate the time windows of significant main effects based on a cluster permutation test.

The ERP results were confirmed by a cluster permutation test (Fig. 6), showing significant category effects from 152 to 260 ms (cluster-based *p* = 0.001) and significant MSS effects from 188 to 292 ms (cluster-based *p* = 0.002). No interaction effects were found in this analysis.

#### Decoding results

**Figure 7.**
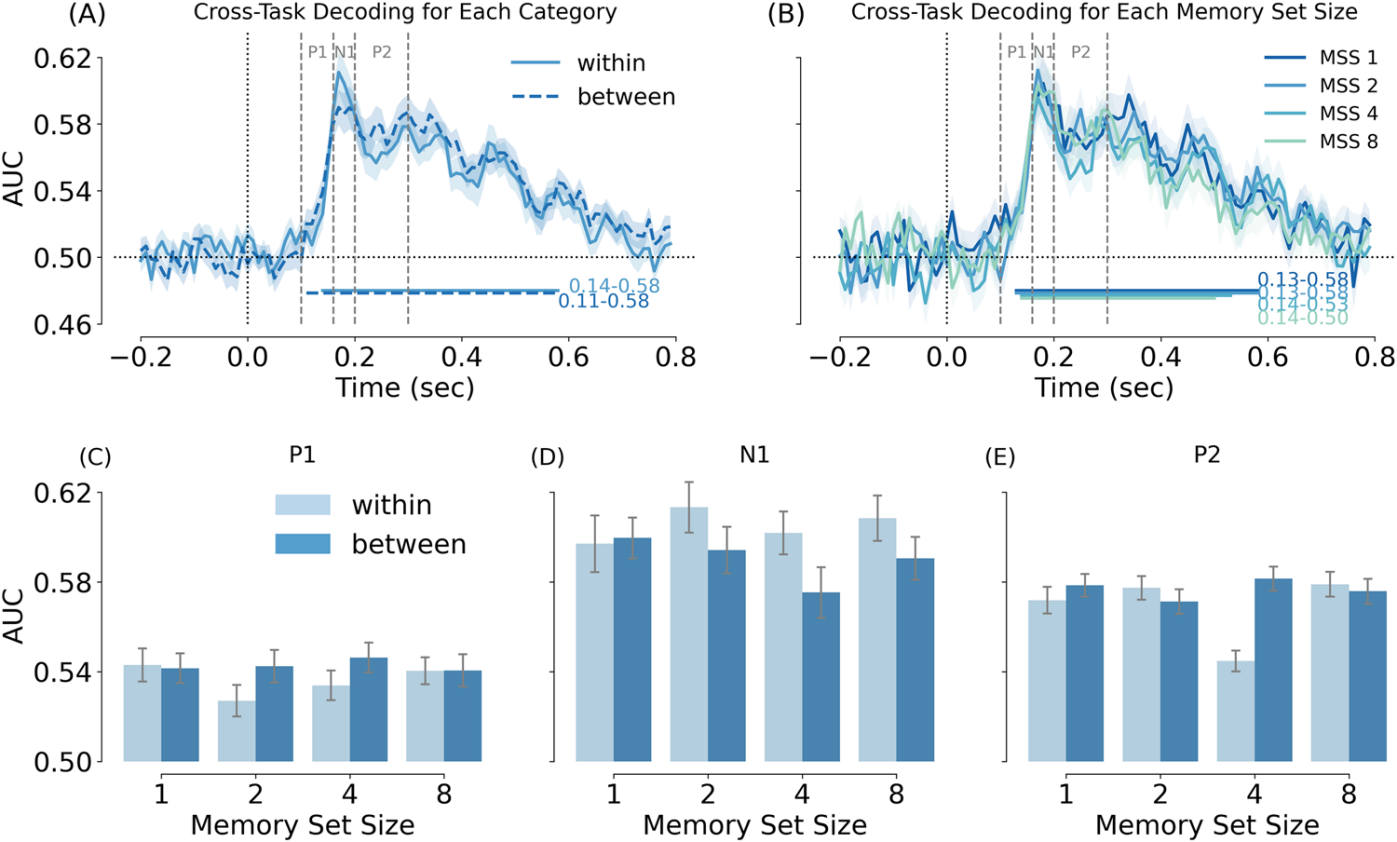
Cross-task decoding in Experiment 1b. ***A***, AUC as a function of category condition. The light blue solid line represents the within-category condition, while the dark blue dashed line represents the between-category condition. ***B***, AUC as a function of MSS. The blue color from dark to light refers to set size 1, 2, 4, and 8, respectively. ***C–E***, Compare the mean AUC in the two categories and four MSS conditions, averaged across the time points of each component (P1, N1, P2). The main effect of category was significant for the N1 time window (panel ***D***).

To test whether attention modulated information about object category, we decoded the category (animate/inanimate) of the distractor objects using a classifier trained on data from a separate experiment that did not involve memory search (see Materials and Methods), following the cross-decoding approach of a previous attention study (Kaiser et al., 2016). Decoding accuracy reflects the representational strength of the distractor objects, rather than the amplitude of the evoked responses. Results showed that AUC scores in all eight conditions reached significance (cluster-based *p* < 0.05) from ∼130–150 to 400–580 ms (Fig. 7*A,B*), with the first peak at ∼170 ms, in line with previous decoding studies (Carlson et al., 2013; Cichy et al., 2014; Kaiser et al., 2016).

Next, we averaged decoding accuracy across the time window of each ERP component and tested these using repeated-measures ANOVAs. In line with the ERP results, no significant effects were observed for the P1 time window (*p* > 0.335, for all tests; Fig. 7*C*). Interestingly, the N1 showed a significant main effect of category, *F*_(1, 31)_ = 4.516, *p* = 0.042, *η_P_*^2^ = 0.127, with better decoding for within- than between-category distractors (Fig. 7*D*). It should be noted, however, that this analysis was not corrected for multiple comparisons. The main effect of MSS and the interaction between MSS and category were not significant (*p* > 0.678, for all tests). Finally, no significant effects were observed for the P2 time window (*p* > 0.109, for all tests; Fig. 7*E*).

#### Summary

The behavioral results of Experiment 1b replicated those of Experiment 1a, again showing that memory search was more efficient for between- than within-category distractors. The ERP results showed that category membership modulated the visually evoked N1 component (160–200 ms) as well as the subsequent P2 component (200–300 ms), while set size only modulated the P2 component. The cluster permutation test confirmed these results, showing a relatively early category effect, from 152 to 260 ms, while set size effects emerged from 188 to 292 ms. Finally, the decoding results of the N1 window showed that object category decoding was higher for within-category distractors than between-category distractors. Altogether, these results provide evidence for category-level attentional modulation during a memory search task. Distractor objects matching the category of the memory set received more processing than nonmatching distractor objects, demonstrated both by a differential evoked responses (VanRullen and Thorpe, 2001) and more accurate categorical representation (Kaiser et al., 2016) ∼160–200 ms after onset.

### Experiment 2

In Experiment 2, we followed up on the findings of Experiment 1b, testing whether category-matching distractors attract spatial attention. Spatial attention shifts were expected to occur later than the category-based modulation of visual processing observed in Experiment 1b, with category-based modulation guiding spatial attention (Battistoni et al., 2020). For example, in a categorical visual search task, category-based modulation of visual object processing (as also observed in Experiment 1b) occurred from 180 ms after image onset (Kaiser et al., 2016) while spatial attention shifts to the target occurred at 240 ms after image onset (Battistoni et al., 2020). By having participants search for targets in two-object displays (Fig. 3), here we could measure the allocation of spatial attention using the lateralized N2pc EEG component, occurring 200–300 ms after image onset: previous studies showed that template-matching objects (e.g., targets) during visual search attract spatial attention, eliciting an N2pc (Eimer, 1996; Luck et al., 2000). This target-elicited N2pc is reduced when a target appears together with a distractor that partially matches the template (Nako et al., 2016; Wu et al., 2016; Yeh et al., 2019; Yeh and Peelen, 2022). Therefore, if participants adopted a category-level attentional template in our memory search task, we expected the N2pc to be reduced when a target appeared next to a within-category (“T-Dw”) as compared to a between-category (“T-Db”) distractor. For the same reason, we expected to observe an N2pc in response to a within-category distractor (“Dw”) when shown together with a between-category (“Db”) distractor.

#### Behavioral results

**Figure 8.**
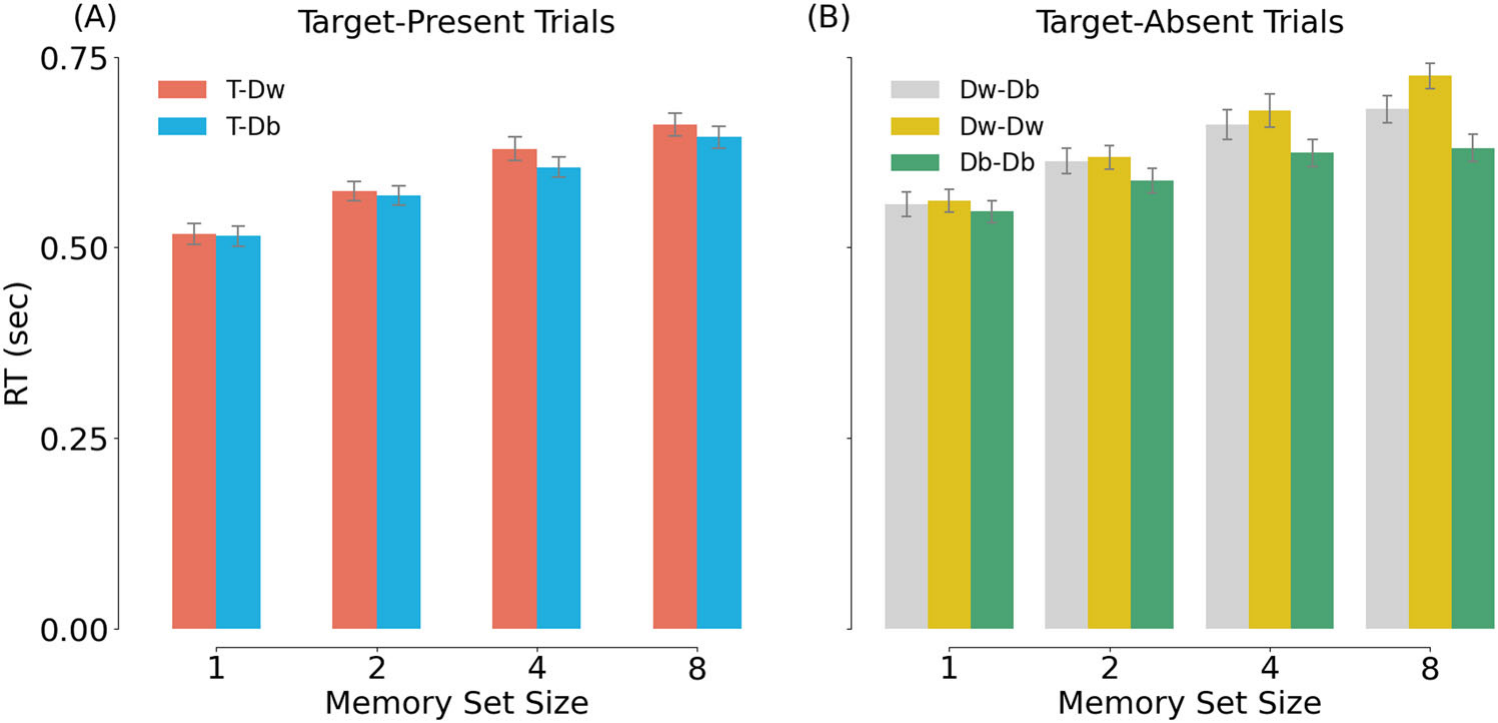
Mean RT (in sec) in Experiment 2. ***A***, RTs in target-present trials. The red bars represent the within-category condition (T-Dw; target shown next to a within-category distractor), while the blue bars represent the between-category condition (T-Db; target shown next to a between-category distractor). ***B***, RTs in target-absent trials. The grey, yellow, and green bars represent Dw-Db, Dw-Dw, and Db-Db trials.

Figure 8*A* shows the RT results for target-present trials. A two-way repeated-measures ANOVA (MSS; 1/2/4/8 × category; T-Dw/T-Db) showed a main effect of MSS, *F*_(2.03, 62.88)_ = 104.097, *p* < 0.001, *η_P_*^2^ = 0.771; a main effect of category, *F*_(1, 31)_ = 32.212, *p* < 0.001, *η_P_*^2^ = 0.510; and an interaction between MSS and category, *F*_(3, 93)_ = 4.568, *p* < 0.001, *η_P_*^2^ = 0.128. Simple main effects of MSS were also significant within both the T-Dw condition, *F*_(2.23, 69.1)_ = 88.3, *p* < 0.001, *η_P_*^2^ = 0.576, and the T-Db condition, *F*_(1.93, 59.9)_ = 92.5, *p* < 0.001, *η_P_*^2^ = 0.749. Simple main effects of category were observed in MSS 4 and 8, *F*_(1, 31)_ = 20.4, *p* < 0.001, *η_P_*^2^ = 0.397 and *F*_(1, 31)_ = 11.5, *p* = 0.002, *η_P_*^2^ = 0.271, but not in MSS 1 and 2, *F*_(1, 31)_ = 0.65, *p* = 0.426, *η_P_*^2^ = 0.021 and *F*_(1, 31)_ = 1.55, *p* = 0.222, *η_P_*^2^ = 0.048.

For target-absent trials (Fig. 8*B*), a two-way repeated-measures ANOVA (MSS 1/2/4/8 × Dw-Db/Dw-Dw/Db-Db category) showed a main effect of MSS, *F*_(3,93) _= 99.905, *p* < 0.001, *η_P_*^2^ = 0.763, a main effect of category, *F*_(1.61,50.05) _= 88.455, *p* < 0.001, *η_P_*^2^ = 0.740, and an interaction between set size and category, *F*_(3.7,114.82) _= 12.108, *p* < 0.001, *η_P_*^2^ = 0.281. Significant main effects of MSS were observed in all three category conditions (Dw-Db, *F*_(3, 93)_ = 95.57, *p* < 0.001, *η_P_*^2^ = 0.755; Dw-Dw, *F*_(3, 93)_ = 75.906, *p* < 0.001, *η_P_*^2^ = 0.710; Db-Db, *F*_(3, 93)_ = 34.368, *p* < 0.001, *η_P_*^2^ = 0.526).

#### ERP results

**Figure 9.**
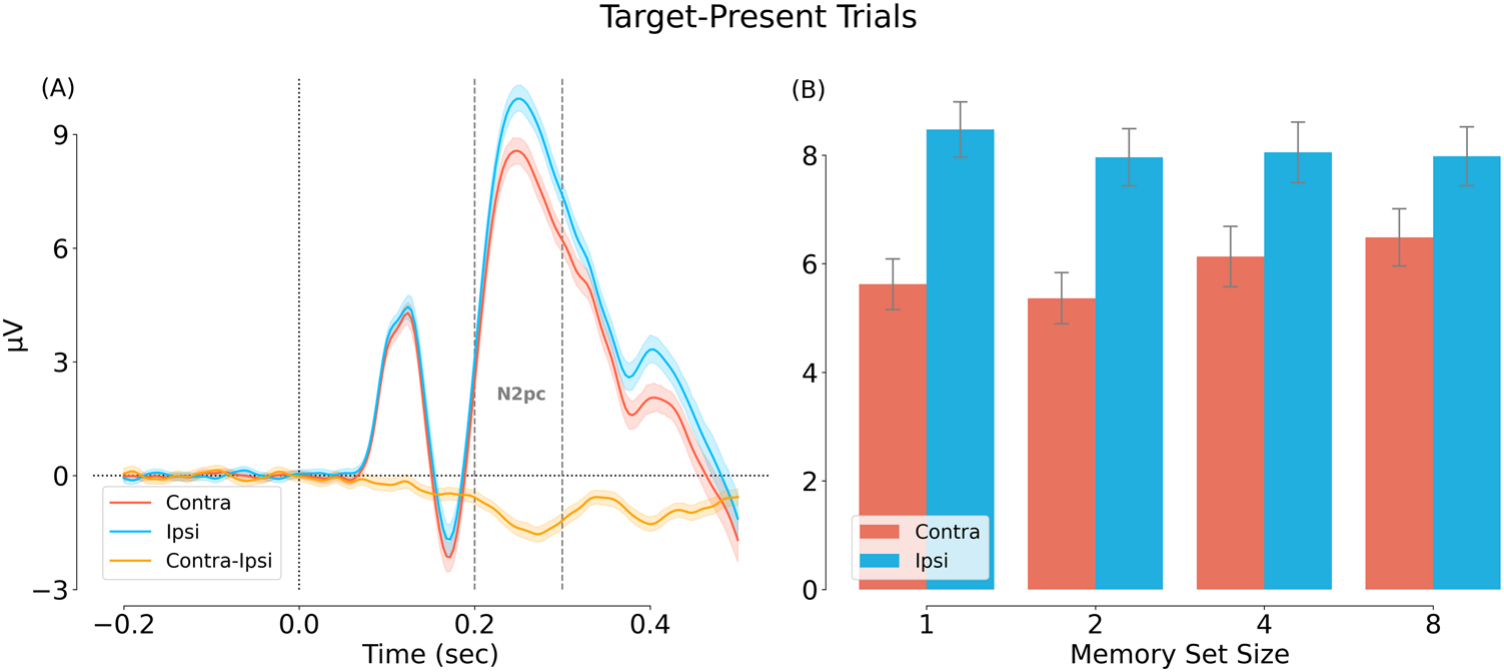
Main effect of visual field location, showing a target related N2pc. ***A***, Main effect of contra/ipsi-lateral visual field. ***B***, Comparison of the contra/ipsi-lateral amplitude averaged across the N2pc time window (200–299 ms).

*N2pc induced by targets*. In the first analysis, we wanted to verify that the targets in our experiment evoked a reliable N2pc. Averaged across conditions, we observed a strong N2pc, with a more negative response contralateral versus ipsilateral to the target from ∼200 ms after stimulus onset (Fig. 9*A*). Next, we averaged the amplitude of the evoked response in the N2pc time window (200–299 ms after onset) and tested the N2pc effect for each set size (Fig. 9*B*). This analysis revealed a significant N2pc for each set size (*p* < 0.005, for all tests).

**Figure 10.**
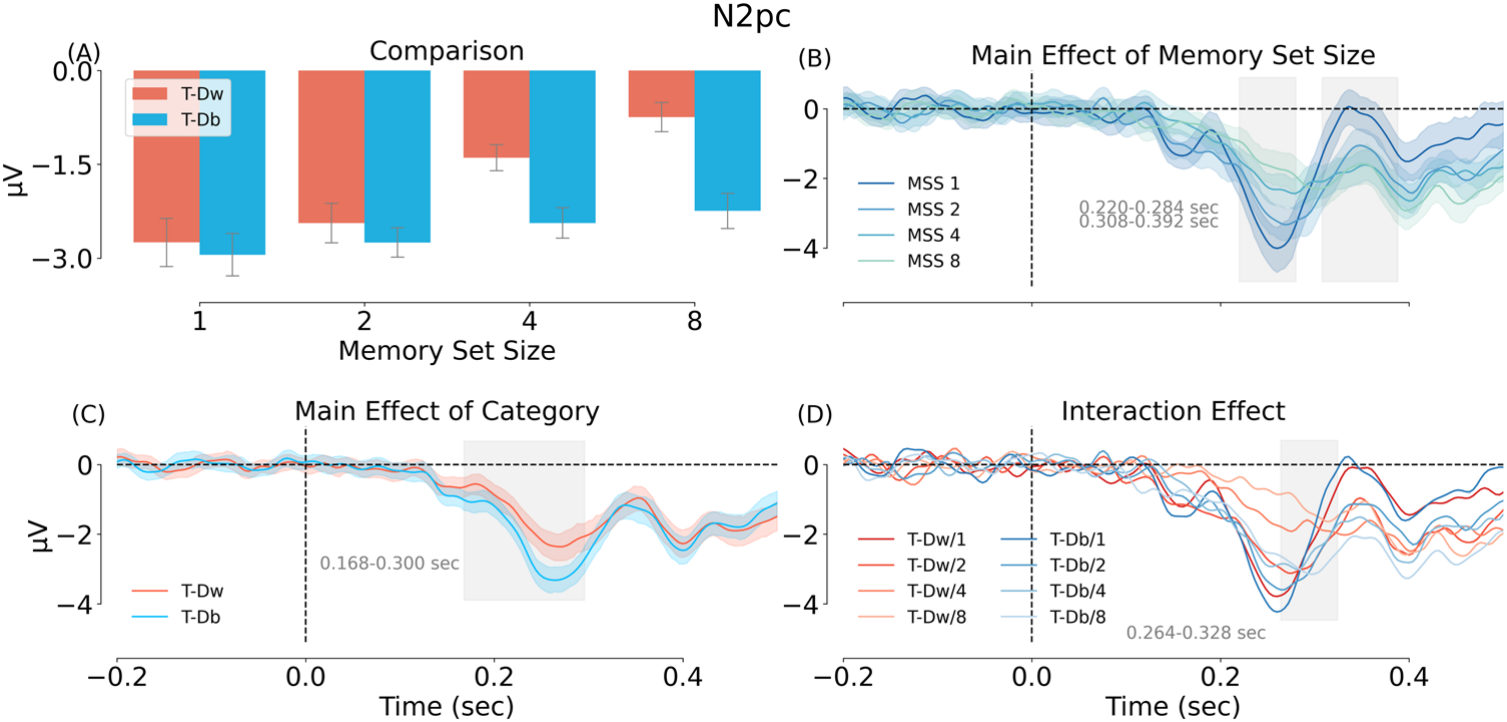
ERP results of Experiment 2. ***A***, Comparison of the mean N2pc amplitude in the two categories and four MSS conditions. ***B***, Main effect of MSS in a cluster analysis. ***C***, Main effect of category in a cluster analysis. ***D***, Interaction of MSS and category in a cluster analysis. Different colors from dark to light refer to MSS 1–8. T-Dw = a target shown next to a within-category distractor, T-Db = a target shown next to a between-category distractor.

Having established a reliable target-related N2pc, we then tested how this effect was modulated by MSS and category through a two-way repeated-measures ANOVA with the N2pc as dependent variable (contra-ipsi, averaged across 200–299 ms) and MSS (1/2/4/8) and category (T-Dw/T-Db) as independent variables. Results are shown in Figure 10*A*. The main effects of MSS and category were significant: MSS, *F*_(3, 93)_ = 8.672, *p* < 0.001, *η_P_*^2^ = 0.219, and category *F*_(1, 31)_ = 49.580, *p* < 0.001, *η_P_*^2^ = 0.615. The interaction between MSS and category was also significant, *F*_(3, 93)_ = 4.822, *p* = 0.004, *η_P_*^2^ = 0.135. Following up on the interaction, we found that the simple main effect of MSS was significant in the T-Dw condition, *F*_(3, 93)_ = 11.4, *p* < 0.001, *η_P_*^2^ = 0.268, but not in the T-Db condition, *F*_(3, 93)_ = 1.87, *p* = 0.14, *η_P_*^2^ = 0.057. Furthermore, the effect of category was significant for MSS 4 and 8, *F*_(1, 31)_ = 20.1, *p* < 0.001, *η_P_*^2^ = 0.394 and *F*_(1, 31)_ = 19.1, *p* < 0.001, *η_P_*^2^ = 0.381, but not for MSS 1 and 2, *F*_(1, 31)_ = 0.64, *p* = 0.43, *η_P_*^2^ = 0.02 and *F*_(1, 31)_ = 1.81, *p* = 0.188, *η_P_*^2^ = 0.055.

These results were confirmed by cluster permutation tests (Fig. 10*B,C*), which showed significant MSS effects from 220 to 284 ms and 308 to 392 ms (both cluster-based *p* = 0.001) and significant category effects from 168 to 300 ms (cluster-based *p* = 0.001). The interaction of the two effects was significant from 264 to 328 ms (cluster-based *p* = 0.013).

Together, these results confirm our first hypothesis, that the target-induced N2pc is reduced in the presence of a within-category distractor. Mirroring the behavioral results, this reduction was stronger for larger set size.

**Figure 11.**
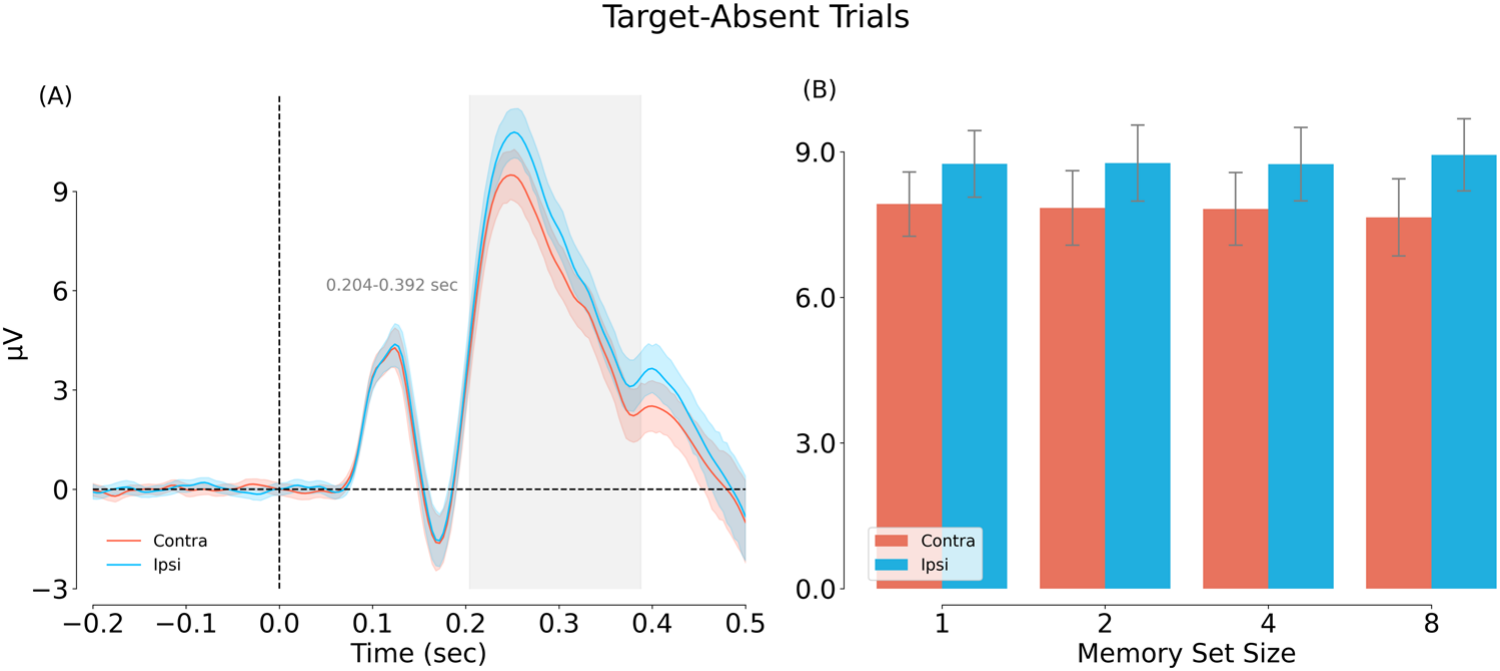
Main effect of visual field location for the Dw-Db condition (target-absent trials). In this analysis, contralateral is relative to the location of the within-category distractor (Dw). ***A***, Main effect of contra/ipsi-lateral visual field based on a cluster permutation test (cluster-based *p* = 0.001). ***B***, Comparison of the contra/ipsi-lateral amplitude averaged across the N2pc time window (200–299 ms).

*N2pc induced by distractors*. Next, we tested our second hypothesis that of an N2pc induced by within-category (“Dw”) versus between-category (“Db”) distractors. Dw-Dw and Db-Db trials were not included in the analysis because there was, by definition, no lateralized attentional bias in these two conditions. Results confirmed our hypothesis: we observed a significant difference between contra- and ipsi-lateral responses from ∼200 ms after stimulus onset (Fig. 11*A*). Averaging responses across the N2pc time window (200 and 299 ms) revealed a significant N2pc: *F*_(1, 31)_ = 121.917, *p* < 0.001, *η_P_*^2^ = 0.797. The N2pc did not differ significantly across set size: *F*_(3, 93)_ = 1.474, *p* = 0.227, *η_P_*^2^ = 0.045 (Fig. 11*B*).

#### Summary

The results of Experiment 2 showed that the target-elicited N2pc was reduced when the target was shown next to a same-category distractor. Furthermore, spatial attention (indexed by the N2pc component) was directed toward distractors that matched the category of the memory set. Altogether, these results provide evidence that participants formed a categorical attentional template, with spatial attention being directed to distractor objects belonging to the category of the memory set.

## Discussion

In three experiments, we investigated the role of attention in memory search. In an online behavioral experiment (Experiment 1a), participants memorized target objects; then, during a memory search phase, they viewed one object at a time and decided whether the object was part of the memorized set of objects. The memory set always consisted of objects from a single category (animate or inanimate objects), while the distractor objects could be from either category. Our analyses focused on responses to these distractor objects (80% of trials) as a function of MSS (1, 2, 4, 8, 16) and category (same or different category as the memory set). Results showed that memory search was much more efficient for between-category than within-category distractors, replicating earlier work (Cunningham and Wolfe, 2012, 2014; Drew and Wolfe, 2014). Using EEG (Experiment 1b), we tested whether this increased efficiency could be explained by attentional modulation at the level of object category. Results confirmed this hypothesis, showing that early visual object processing was modulated by the category membership of the distractor: We found a larger N1 in response to distractors from the target category compared to distractors from a different category. Furthermore, decoding analyses showed that within-category distractors were more strongly represented than between-category distractors at this latency. The results of Experiment 1 are in line with the attentional modulation of visual processing observed in single-target detection tasks (VanRullen and Thorpe, 2001; Kaiser et al., 2016). This modulation is much earlier than the typical time window of memory retrieval, which starts after ∼300 ms (Rugg et al., 1998; Curran and Hancock, 2007; Rugg and Curran, 2007; Noh et al., 2018). In Experiment 2, we presented two objects simultaneously to test whether spatial attention (indexed by the N2pc component) was guided to the location of template-matching objects (Hopf et al., 2004; Eimer, 2014; Battistoni et al., 2020; Wolfe, 2021). We found that spatial attention was directed toward distractor objects that were of the same category as the items in the memory set. Together, the results provide evidence that participants spontaneously used the shared category of the memory items to form a category-level attentional template. By allocating more attentional resources to the features (N1) and location (N2pc) of the target category, they were able to efficiently reject between-category distractors before commencing search in long-term memory.

The behavioral results of all experiments (Figs. 4, 8) and the target-related N2pc results of Experiment 2 (Fig. 10A) revealed that category interacted with MSS, such that the categorical modulation became stronger with increasing set size. This result can be explained in at least two ways. First, it is possible that participants only started to use an attentional template-based strategy when memory search became effortful (i.e., with high set size). Alternatively, participants may have adopted an attentional template-based strategy for all set sizes, but the specificity of the template varied with the MSS. For a set size of two, the template may have been specific to the subcategories of the two targets (e.g., a cat and a horse). In that case, distractor objects from other subcategories (e.g., reptile, bird) may not have provided a strong match to the template. Instead, for higher set sizes, a larger number of subcategories made up the memory set. In that case, the features that the subcategories had in common were more likely to generalize to the distractor objects from the same superordinate category (e.g., animals). Future studies could systematically manipulate the similarity of the subcategories within the memory set to distinguish between these accounts.

The interaction between set size and category also shows that set size more strongly affected the rejection of within-category than between-category distractors. This suggests that within-category distractors activated a memory search process, while between-category distractors did so only weakly. Of note, the effect of set size was still significant for between-category distractors in all behavioral analyses, suggesting that categorical attention was not fully preventing between-category distractors from entering the memory search process.

Interestingly, not all analyses showed an interaction between set size and category. Specifically, the modulation of visual distractor processing in Experiment 1b (Figs. 5*C*, 7*D*) and the distractor evoked N2pc in Experiment 2 (Fig. 11*B*) only showed a main effect of category. It is possible that the absence of an interaction in these analyses reflected a lack of power (e.g., the weak trend toward an interaction in Fig. 11*B*). Alternatively, the interaction may reflect a true dissociation between these measures. For example, in Experiment 2, if we assume that the within-category distractor only weakly matched the template when set size was low (e.g., because the template was specific to the subcategories in the memory set) these distractors would not have provided strong competition when shown next to a target, resulting in the category × set size interaction observed for the target-related N2pc. However, when the same within-category distractor is shown next to a between-category distractor, it would still provide a relatively better match to the template than the between-category distractor, and thus attract spatial attention even in the low set size conditions.

Our findings raise the question of what features the categorical template consists of, and whether categorical templates are specific to the categories used here. Animate and inanimate objects differ in terms of mid-, and high-level visual features (e.g., Proklova et al., 2016; Long et al., 2018; Thorat et al., 2019; Jozwik et al., 2022), and it has been proposed that the human visual system is particularly sensitive to these category-diagnostic features (New et al., 2007), as also reflected in the animate–inanimate organization of the ventral visual cortex (Chao et al., 1999; Kriegeskorte et al., 2008; Grill-Spector and Weiner, 2014; Thorat et al., 2019). This raises the possibility that categorical attention in memory search, as revealed here, is specific to the distinction between animate and inanimate objects. Future studies will need to test whether our results generalize to other categorical distinctions (e.g., fruit vs nonfruit). We anticipate that results are most likely to generalize to categories that, like animals, are highly familiar and that are characterized by diagnostic visual features (Battistoni et al., 2017).

### Conclusion

To conclude, our study reveals a crucial role of attention in memory search. When observers look for multiple objects at the same time, they can use the objects’ shared categorical features to direct attention at that level, leading to the efficient rejection of distractor objects belonging to other categories (Fig. 1*A*).

## Synthesis

Reviewing Editor: Ifat Levy, Yale School of Medicine Decisions are customarily a result of the Reviewing Editor and the peer reviewers coming together and discussing their recommendations until a consensus is reached. When revisions are invited, a fact-based synthesis statement explaining their decision and outlining what is needed to prepare a revision will be listed below. The following reviewer(s) agreed to reveal their identity: Sean Noah. Note: If this manuscript was transferred from JNeurosci and a decision was made to accept the manuscript without peer review, a brief statement to this effect will instead be what is listed below.

The authors have adequately responded to reviewers' comments.
